# Association of inflammatory markers and multimorbidity in young adults: cross-sectional findings from the Pelotas (Brazil) birth cohort, 1993

**DOI:** 10.1590/0102-311XEN191623

**Published:** 2025-02-07

**Authors:** Isabel Oliveira de Oliveira, Tulio L. Correa, Gustavo Dias Ferreira, Bruno Pereira Nunes, Helen Gonçalves, Fernando César Wehrmeister, Ana Maria Baptista Menezes

**Affiliations:** 1 Instituto de Biologia, Universidade Federal de Pelotas, Pelotas, Brasil.; 2 Faculdade de Medicina, Universidade Federal de Pelotas, Pelotas, Brasil.; 3 Escola Superior de Educação Física e Fisioterapia, Universidade Federal de Pelotas, Pelotas, Brasil.; 4 Faculdade de Enfermagem, Universidade Federal de Pelotas, Pelotas, Brasil.

**Keywords:** Gender and Health, Gender-inclusive Policies, Evidence-informed Policies, Saúde e Gênero, Políticas Inclusivas de Gênero, Política Informada por Evidências, Género y Salud, Políticas Inclusivas de Género, Política Informada por la Evidencia

## Abstract

Early diagnosis can be a beneficial factor for minimizing health risks related to multimorbidity. This study aims to assess the association of multimorbidity with interleukin 6 (IL-6), C-reactive protein (CRP), and adiponectin in 22-year-old participants of the Pelotas (Brazil) birth cohort. A total of 3,578 subjects had serum measurements of IL-6, CRP, and adiponectin at the 22-year-old visit. For multimorbidity evaluation, a list of 15 morbidities was used and divided into subgroups (cardiometabolic, pulmonary, allergic diseases, and mental disorders). The occurrence of ≥ 2 morbidities was higher in females (55.1%) than in males (45.2%). A negative association between multimorbidity and adiponectin was found in females, whereas positive associations between the number of diseases and IL-6 and CRP were observed in males. For both sexes, cardiometabolic problem was the morbidity most associated with the markers. The analysis for isolated diseases identified dyslipidemia was the only cardiometabolic condition associated with physiological markers. Our findings suggest an inverse association between multimorbidity and adiponectin in females, as well as a direct cumulative association between the number of diseases and IL-6 and CRP in males at a young age.

## Resumo

O diagnóstico precoce pode ser um fator benéfico para minimizar os riscos à saúde relacionados à multimorbidade. Este estudo teve como objetivo avaliar a associação da multimorbidade com interleucina 6 (IL-6), proteína C-reativa (CRP) e adiponectina em participantes de 22 anos da coorte de nascimentos de Pelotas, Rio Grande do Sul, Brasil. Ao todo, 3.578 sujeitos passaram por medições séricas de IL-6, CRP e adiponectina na visita dos 22 anos. Para a avaliação da multimorbidade, foi utilizada uma lista de 15 morbidades, dividida em subgrupos (doenças cardiometabólicas, pulmonares, alérgicas e distúrbios mentais). A ocorrência de ≥ 2 morbidades foi maior em mulheres (55,1%) do que em homens (45,2%). Foi encontrada uma associação negativa entre multimorbidade e adiponectina em mulheres, enquanto associações positivas entre o número de doenças e IL-6 e CRP foram observadas em homens. Para ambos os sexos, condição cardiometabólica foi a morbidade mais consistentemente associada aos marcadores. A análise isolada das doenças identificou que a dislipidemia foi a única condição cardiometabólica associada aos marcadores fisiológicos. Os achados sugerem uma associação inversa entre multimorbidade e adiponectina em mulheres, bem como uma associação cumulativa direta entre o número de doenças e IL-6 e CRP em homens jovens.

## Resumen

El diagnóstico precoz puede ser un factor beneficioso para minimizar los riesgos para la salud relacionados con la multimorbilidad. Este estudio tuvo como objetivo evaluar la asociación de multimorbilidad con interleucina 6 (IL-6), proteína C reactiva (PCR) y adiponectina en participantes de 22 años de edad en la cohorte de nacimientos de Pelotas, Rio Grande do Sul, Brasil. La muestra estuvo conformada por 3.578 sujetos de 22 años de edad que tuvieron mediciones en suero de IL-6, PCR y adiponectina. Para evaluar la multimorbilidad, se utilizó una lista de 15 morbilidades, divididas en subgrupos (trastornos cardiometabólicos, pulmonares, alérgicos y mentales). La aparición de ≥ 2 morbilidades fue mayor en mujeres (55,1%) que en hombres (45,2%). Se encontró una asociación negativa entre la multimorbilidad y la adiponectina en las mujeres, mientras que se observaron asociaciones positivas entre el número de enfermedades y la IL-6 y la PCR en los hombres. Para ambos sexos, el problema cardiometabólico tuvo una mayor asociación consistente de morbilidad con los marcadores. El análisis aislado de las enfermedades identificó que la dislipidemia fue la única condición cardiometabólica asociada con los marcadores fisiológicos. Los hallazgos indican una asociación inversa entre la multimorbilidad y la adiponectina en mujeres, así como una asociación acumulativa directa entre el número de enfermedades e IL-6 y PCR en hombres jóvenes.

## Introduction

Multimorbidity is the occurrence of two or more diseases in the same patient and is related to decreased quality of life and increased mortality risk ^1^ ^,^ ^2^ ^,^ ^3^. Prevalence of multimorbidity was 33% in a meta-analysis including 68 studies from different populations of 18 years or over ^4^. On the Brazilian population, a 22% prevalence was described in a study performed with a national representative sample, with females showing 1.9% more odds of multimorbidity than males ^5^. Because a rapid aging of the world population has been observed, multimorbidity has become an important public health concern, as it substantially increases with age ^4^. Impacting various health services from general practice to end-of-life care, multimorbidity is a challenge to healthcare systems ^6^. Lower socioeconomic status, physical activity, higher body mass index (BMI) and smoking have also been associated with increased risk of multimorbidity ^7^ ^,^ ^8^ ^,^ ^9^.

Early diagnosis of multimorbidity can be a beneficial factor for managing this condition and minimizing health risks ^10^. An alternative could be to establish associated specific inflammatory markers, but this has been a challenge for health services ^11^. Some initial efforts indicated that lower levels of dehydroepiandrosterone sulfate (DHEAS) and elevated levels of interleukin 6 (IL-6), C-reactive protein (CRP), lipoprotein (Lp), and cystatin C (Cyst-C) may be associated with multimorbidity ^12^. Still, most studies published on this subject were performed on older adults. However, it is crucial to investigate the characteristics of multimorbidity in young adults to better understand this condition development.

Furthermore, inflammatory markers such as CRP and IL-6 are positively associated with obesity ^13^, while adiponectin levels are low in individuals with obesity ^14^. Obesity-related conditions include heart disease, stroke, type 2 diabetes, and some types of cancer, which are among the leading preventable diseases in the world and contribute to multimorbidity ^15^ ^,^ ^16^ ^,^ ^17^. A cumulative association between an increase in IL-6 and CRP and a decrease in adiponectin in patients with higher adiposity ^18^ ^,^ ^19^ and lower bone mineral density ^20^ was previously shown at 22-year-old individuals by our research group.

Therefore, this study aims to assess the associations between multimorbidity and IL-6, CRP, and adiponectin in 22-year-old participants of the 1993 Pelotas (Brazil) birth cohort, stratified by sex. Also, we aim to evaluate physiological markers associated with disease accumulation in young adults, hypothesizing that individuals with multimorbidity have elevated pro-inflammatory cytokines even at a young age.

## Methods

During 1993, the five maternity hospitals of the city of Pelotas, Rio Grande do Sul State, Brazil, were visited daily, and all mothers of live-born babies were invited to take part in a prospective study. Of the 5,265 recorded births in the city that year, 5,249 agreed to participate in the longitudinal study ^21^. After delivery, the mothers were interviewed about their pregnancy and health-related variables and the newborns had their anthropometric measurements performed ^22^. Sub-samples of this cohort were followed-up during childhood. Follow-ups of the full cohort took place at 11, 15, 18, and 22 years ^23^ ^,^ ^24^. The 22-year follow-up occurred from October 2015 to July 2016, when interviews and clinical measurements took place, and non-fasting blood samples were drawn from participants. Pregnancy was the exclusion criteria for collecting blood. All samples were processed in the laboratory and stored in freezers with ultra-low temperature.

This analysis included all participants who had serum measurements of IL-6, CRP, and adiponectin at the 22-year follow-up with known fasting status. IL-6 was evaluated by the Quantikine HS Human IL-6 immunoassay kit (R&D Systems Inc., https://www.rndsystems.com/), CRP was measured by immunoturbidimetric assay (Labtest Diagnóstica SA, https://labtest.com.br/), and adiponectin concentration was determined by ELISA Quantikine Human Total Adiponectin Immunoassay kit (R&D Systems Inc.). The intra and inter-assay coefficients of variation of the tests were, respectively: 4.1% and 13.4% for IL-6, 2% and 2.1% for CRP, and 9.1% and 13.2% for adiponectin. The results are expressed in pg/mL for IL-6, mg/L for CRP, and µg/mL for adiponectin.

For multimorbidity evaluation, a list of 15 morbidities was used and divided into the following groups: cardiometabolic diseases (diabetes, high blood pressure, and dyslipidemias); pulmonary diseases (asthma and lung problem); allergic diseases (allergic rhinitis, allergic conjunctivitis, and eczema); and mental disorders (depression, generalized anxiety disorder, attention deficit hyperactivity disorder, social phobia, antisocial personality disorder, bipolar disorder, and post-traumatic stress disorder). In this study, individuals showing two or more of the morbidities above were considered as having multimorbidity. The question “Has any physician diagnosed you as having (each disorder)?”, was asked to evaluate the following diseases based on self-reported medical diagnosis: cardiometabolic diseases, asthma, and allergic diseases. Dyslipidemias were evaluated based on the cardiovascular prevention guidelines of the Brazilian Society of Cardiology ^25^ considering the reference values of ≥ 160mg/dL for low-density lipoprotein, ≥ 175mg/dL for triglycerides, and/or high-density lipoprotein of < 40mg/dL for males and < 50mg/dL for females. Based on the *ESC/ESH Guidelines for Management of Arterial Hypertension* ^26^, reference values to classify high blood pressure were systolic blood pressure ≥ 140mmHg and/or diastolic blood pressure ≥ 90mmHg. Spirometry with a forced expiratory volume in one second (FEV1)/forced vital capacity (FVC) relation < 85% of the predicted value was used to classify lung problem ^27^. Females who had high blood pressure or diabetes only during pregnancy were not considered to have these diseases. Prevalence of mental disorders [major depressive disorder (MDD), generalized anxiety disorder (GAD), social anxiety disorder (SAD), attention-deficit/hyperactivity disorder (ADHD), bipolar disorders type 1 and 2 (BD1, BD2), post-traumatic stress disorder (PTSD), and antisocial personality disorder (APD)] and suicide risk were assessed using the *Mini International Neuropsychiatric Interview* ^28^.

The covariables considered were: skin color (white, black, and other); asset index at 22 years (in quintiles); harmful alcohol intake ^29^ (*Alcohol Use Disorder Identification Test* - AUDIT ≥ 8 points: no, yes); smoking status (none, > 0 to 1 cigarette/day, > 1 cigarette/day); physical activity measured via a standardized and previously tested questionnaire ^30^ (inactive: < 150 minutes per week, active: ≥ 150 minutes per week); trunk fat mass (%, dual-energy X-ray absorptiometry - DXA); difference of time between blood drawing and last meal; and the use of oral contraceptives. Sociodemographic and behavioral data were obtained by questionnaires. Adiposity was measured by DXA model Lunar Prodigy Advance Bone Densitometer (GE Healthcare, https://www.gehealthcare.com/).

To display descriptive analyses, absolute and relative frequencies were used for categorical variables. The continuous variables IL-6 and CRP were converted into logarithms, and their adequations were verified via residual analyses. The Supplementary Material (https://cadernos.ensp.fiocruz.br/static//arquivo/supl-e00191623_6545.pdf) shows the distributions of these two variables. The results were described as exponential median with its 25-75 interquartile range (25-75 IQR). Adiponectin was reported as mean and 95% confidence interval (95%CI). Crude and adjusted linear regressions have been performed to analyze the associations. Results were expressed as β coefficients (95%CI) or exponential means (95%CI). Analyses were sex-stratiﬁed due to interactions between multimorbidity, sex, and adiponectin (p = 0.130). The Stata software, version 13 (https://www.stata.com/) was used for the analyses. In the Wald test for linear regression or heterogeneity, values of p < 0.05 were considered statistically significant. To minimize type I error, which may result from multiple statistical comparisons, Bonferroni correction was applied to the original p-value < 0.05.

The Federal University of Pelotas Research Ethics Committee approved the cohort follow-ups projects. For the 22-year follow-up of the Pelotas cohort, the approval protocol was 1,250,366. All cohort participants or their caregivers signed an informed consent form and the dataset was anonymized.

## Results

The response rate of the 1993 Pelotas (Brazil) birth cohort, for the 22-year follow-up was 76.3% of the original cohort. The total sample size for this analyses was composed of 3,482 participants (53.7% females) of approximately 22 years old. Table 1 shows descriptive characteristics of the sample. Most males and females reported white skin color, and males were wealthiest than females. Regarding lifestyle: males smoked more, had a higher harmful alcohol intake, and were more physically active compared to females.

**Table 1:** Sample description (individuals with interleukin 6 - IL-6, C-reactive protein - CRP, and/or adiponectin information) according to demographic, socioeconomic, and health problems, stratified by sex, at 22 years old (n = 3,482).

Parameter	Total	Male	Female
	n (%)	n (%)	n (%)
Skin color	3,301	1,560	1,741
White	2,082 (63.1)	992 (63.6)	1,090 (62.6)
Black	503 (15.2)	232 (14.9)	271 (15.6)
Others	716 (21.7)	336 (21.5)	380 (22.8)
Asset index (quintiles)	3,477	1,659	1,818
1st	699 (20.1)	271 (16.3)	428 (23.5)
2nd	690 (19.8)	304 (18.3)	386 (21.2)
3rd	706 (20.3)	340 (20.6)	366 (20.1)
4th	697 (20.1)	369 (22.2)	328 (18.1)
5th	685 (19.7)	375 (22.6)	310 (17.1)
Number of diseases	3,167	1,509	1,658
0	598 (18.9)	342 (22.7)	256 (15.4)
1	973 (30.7)	485 (32.1)	488 (29.5)
2	794 (25.1)	374 (24.8)	420 (25.3)
3	446 (14.1)	181 (12.0)	265 (16.0)
4	226 (7.1)	83 (5.5)	143 (8.6)
5+	130 (4.1)	44 (2.9)	86 (5.2)
Cardiometabolic problems	3,458	1,650	1,808
Yes	1,848 (53.4)	862 (52.2)	986 (54.5)
Respiratory problems	3,213	1,530	1,683
Yes	943 (29.3)	448 (29.3)	495 (29.4)
Allergic problems	3,453	1,648	1,805
Yes	1,208 (35.0)	455 (27.6)	753 (41.7)
Mental disorders	3,459	1,647	1,812
Yes	795 (23.0)	285 (17.3)	510 (28.1)
Smoking status	3,478	1,660	1,818
Never smoker	2,535 (72.9)	1,149 (69.2)	1,386 (76.3)
Former smoker	351 (10.1)	167 (10.1)	184 (10.1)
Current smoker	592 (17.0)	344 (20.7)	248 (13.6)
Harmful alcohol intake	3,477	1,659	1,818
No	2,715 (78.1)	1,158 (69.8)	1,557 (85.6)
Yes (AUDIT > 8 points)	762 (21.9)	501 (30.2)	261 (14.4)
Total physical activity *	3,472	1,655	1,817
Inactive	1,183 (34.1)	421 (25.4)	762 (41.9)
Active	2,289 (65.9)	1,234 (74.6)	1,055 (58.1)
Fat mass index DXA [mean (95%CI)]	3,225	20.8 (20.3; 21.3)	38.0 (37.5; 38.4)
IL-6 (pg/mL) [median (25-75 IQR)]	3,138	1.1 (0.8; 1.7)	1.3 (0.8; 2.0)
CRP (mg/dL) [median (25-75 IQR)]	3,481	0.6 (0.3; 1.4)	1.6 (0.6; 4.1)
Adiponectin (µg/mL) [mean (95%CI)]	3,481	8.0 (7.8; 8.2)	10.7 (10.5; 10.9)

95%CI: 95% confidence interval; AUDIT: *Alcohol Use Disorder Identification Test*; DXA: dual-energy X-ray absorptiometry; IQR: interquartile range.

* Classified as inactive those who do not reach 150 minutes per week of physical activities in leisure time.

The occurrence of ≥ 2 morbidities was higher in females (57.3%) than in males (42.7%). For both sexes, the most prevalent morbidity was cardiometabolic problems (54.5% in females and 52.2% in males). Almost one third of the whole sample presented respiratory problems. Females also had more allergic (41.7% vs. 27.6%) and mental disorders (28.1% vs. 17.3%) than males. Regarding cardiometabolic problems, statistically significant differences were found for hypertension and dyslipidemia in males compared to females (28.2% vs. 12.1% and 34.6% vs. 47.8%, respectively), but not for diabetes (4.1% vs 4.6%) (data not shown). The median (25-75 IQR) for IL-6 (pg/mL) was higher in females than males [1.3 (0.9; 2.0) vs. 1.1 (0.8; 1.7)]; the same was observed for CRP (mg/dL) [1.6 (0.6; 4.1) vs. 0.6 (0.3; 1.4)]. The mean (95%CI) for adiponectin (mg/mL) was also higher in females than in males [10.72 (10.50; 10.94) vs. 7.97 (7.78; 8.16)]. Table 2 shows the association between physiological markers and multimorbidity (presence of ≥ 2 morbidities). Although most crude analyses were considered significant, no statistical significance remained for most of them after adjusting for confounders. The exception was for adiponectin in females, which was still significantly reduced with the presence of multimorbidity [11.06 (10.72; 11.39) vs. 10.37 (10.06; 10.67)].

**Table 2:** Physiological markers and presence of multimorbidity in 22-year-old participants stratified by sex.

Physiological marker/Exposure	Male				Female			
	Crude		Adjusted		Crude		Adjusted	
	Mean	95%CI	Mean	95%CI	Mean	95%CI	Mean	95%CI
IL-6 (pg/mL)		p = 0.001		p = 0.109		p = 0.001		p = 0.819
Multimorbidity								
No	1.14	1.09; 1.19	1.15	1.10; 1.20	1.27	1.21; 1.34	1.42	1.35; 1.48
Yes	1.28	1.22; 1.35	1.22	1.16; 1.28	1.44	1.37; 1.50	1.43	1.37; 1.49
CRP (mg/L)		p < 0.001		p = 0.121		p = 0.020		p = 0.325
Multimorbidity								
No	0.63	0.58; 0.68	0.70	0.64; 0.76	1.45	1.32; 1.60	1.85	1.68; 2.03
Yes	0.81	0.74; 0.88	0.77	0.70; 0.84	1.69	1.55; 1.84	1.96	1.80; 2.13
Adiponectin (µg/dL)		p = 0.009		p = 0.247		p < 0.001		p = 0.003
Multimorbidity								
No	8.15	7.88; 8.42	8.01	7.73; 8.29	11.22	10.88; 11.55	11.06	10.72; 11.39
Yes	7.62	7.33; 7.92	7.76	7.45; 8.07	10.22	9.91; 10.52	10.37	10.06; 10.67

95%CI: 95% confidence interval; CRP: C-reactive protein; IL-6: interleukin 6.

Note: multimorbidity (no: 0-1 morbidity, yes: 2 or more morbidities). Adjusted for skin color, asset index at 22 years, fat mass index (dual-energy X-ray absorptiometry - DXA), difference of time between blood drawing and last meal, use of oral contraceptive, and behavioral variables. Regressions performed with IL-6 and CRP on logarithmic scale - results presented as exponential means and adiponectin as mean (95%CI). In bold: p-value < 0.008 was considered statistically significant after Bonferroni correction.

Supplementary Material (https://cadernos.ensp.fiocruz.br/static//arquivo/supl-e00191623_6545.pdf) shows the adjusted associations between markers and morbidities stratified by the number of diseases and by sex. Positive associations with IL-6 and CRP remained significant only in males, whereas an inverse association was observed in both sexes for adiponectin, although it was not statistically significant. 

Table 3 shows the associations between physiological markers and each morbidity group. For cardiometabolic problems, all crude analyses were significant and remained so after adjustment, except for CRP in females. There was no statistical significance for the associations between any measured marker and allergic, respiratory, or mental disorders.

**Table 3:** Physiological markers and each morbidity group.

Physiological marker/Exposure	Male				Female			
	Crude		Adjusted		Crude		Adjusted	
	Coefficient	95%CI	Coefficient	95%CI	Coefficient	95%CI	Coefficient	95%CI
IL-6 (pg/mL)								
Cardiometabolic		p < 0.001		p < 0.001		p < 0.001		p < 0.001
No	1.06	1.01; 1.11	1.08	1.02; 1.13	1.20	1.14; 1.26	1.33	1.27; 1.39
Yes	1.36	1.30; 1.42	1.29	1.23; 1.36	1.54	1.48; 1.61	1.52	1.46; 1.58
Allergic		p = 0.210		p = 0.071		p = 0.046		p = 0.047
No	1.22	1.17; 1.27	1.21	1.16; 1.25	1.42	1.36; 1.48	1.47	1.41; 1.53
Yes	1.16	1.09; 1.24	1.12	1.05; 1.20	1.32	1.26; 1.39	1.38	1.31; 1.45
Respiratory		p = 0.054		p = 0.213		p = 0.011		p = 0.397
No	1.18	1.13; 1.22	1.16	1.12; 1.21	1.32	1.27; 1.38	1.41	1.36; 1.46
Yes	1.26	1.19; 1.34	1.22	1.14; 1.29	1.45	1.37; 1.55	1.45	1.37; 1.53
Mental		p = 0.811		p = 0.588		p = 0.213		p = 0.719
No	1.20	1.16; 1.25	1.19	1.14; 1.23	1.36	1.30; 1.41	1.44	1.39; 1.49
Yes	1.19	1.10; 1.29	1.16	1.06; 1.25	1.42	1.33; 1.51	1.42	1.34; 1.50
CRP (mg/L)								
Cardiometabolic		p < 0.001		p < 0.001		p < 0.001		p = 0.002
No	0.56	0.51; 0.60	0.62	0.56; 0.68	1.38	1.26; 1.51	1.73	1.57; 1.89
Yes	0.89	0.82; 0.97	0.85	0.78; 0.92	1.85	1.71; 2.01	2.08	1.91; 2.25
Allergic		p = 0.504		p = 0.111		p = 0.860		p = 0.354
No	0.72	0.67; 0.77	0.76	0.70; 0.82	1.61	1.49; 1.74	1.97	1.81; 2.14
Yes	0.69	0.62; 0.77	0.68	0.60; 0.76	1.63	1.48; 1.79	1.87	1.69; 2.04
Respiratory		p = 0.004		p = 0.102		p = 0.580		p = 0.785
No	0.66	0.62; 0.71	0.71	0.65; 0.76	1.56	1.45; 1.69	1.92	1.78; 2.0
Yes	0.80	0.72; 0.90	0.79	0.70; 0.88	1.63	1.45; 1.83	1.89	1.68; 2.10
Mental		p = 0.648		p = 0.495		p = 0.482		p = 0.938
No	0.70	0.66; 0.75	0.74	0.69; 0.79	1.64	1.52; 1.76	1.93	1.78; 2.07
Yes	0.73	0.63; 0.84	0.70	0.60; 0.80	1.56	1.39; 1.75	1.92	1.70; 2.14
Adiponectin (µg/dL)								
Cardiometabolic		p < 0.001		p < 0.001		p < 0.001		p < 0.001
No	8.64	8.37; 8.91	8.46	8.16; 8.76	11.95	11.64; 12.26	11.65	11.33; 11.98
Yes	7.35	7.09; 7.62	7.42	7.14; 7.72	9.69	9.40; 9.97	9.83	9.53; 10.12
Allergic		p = 0.786		p = 0.637		p = 0.001		p = 0.010
No	7.98	7.76; 8.21	7.90	7.66; 8.14	10.40	10.12; 10.69	10.42	10.13; 10.71
Yes	7.92	7.56; 8.29	8.02	7.61; 8.43	11.15	10.82; 11.49	11.02	10.68; 11.37
Respiratory		p = 0.940		p = 0.720		p = 0.243		p = 0.620
No	7.90	7.66; 8.13	7.86	7.62; 8.10	10.75	10.49; 11.02	10.64	10.38; 10.91
Yes	7.91	7.55; 8.28	7.94	7.57; 8.32	10.46	10.05; 10.88	10.77	10.35; 11.18
Mental		p = 0.025		p = 0.022		p = 0.569		p = 0.852
No	7.86	7.65; 8.07	7.81	7.58; 8.03	10.75	10.49; 11.00	10.67	10.41; 10.92
Yes	8.44	7.98; 8.90	8.45	7.95; 8.95	10.61	10.20; 11.01	10.62	10.20; 11.04

95%CI: 95% confidence interval; CRP: C-reactive protein; IL-6: interleukin 6.

Note: morbidities were divided into groups (cardiometabolic, allergic, respiratory, and mental group). Adjusted for skin color, asset index at 22 years, fat mass index (dual-energy X-ray absorptiometry - DXA), difference of time between blood drawing and last meal, use of oral contraceptive, and behavioral variables. Regressions performed with IL-6 and CRP on logarithmic scale - results presented as exponential means and adiponectin as mean (95%CI). In bold: p-value < 0.002 was considered statistically significant after Bonferroni correction.

Finally, the associations between the physiological markers and the reported or measured cardiometabolic problems were analyzed (Table 4). The statistical significance for hypertension was lost after adjustment. The associations of dyslipidemia with IL-6 and adiponectin remained significant after adjustment in both sexes. However, a significant adjusted association between CRP and dyslipidemia was observed in males (p < 0.001) but not in females (p = 0.005). Therefore, in the presence of dyslipidemia, males and females had higher IL-6 and lower adiponectin serum concentrations, whereas higher CRP was observed only in males. There was no statistical significance between diabetes and any measured marker in crude or adjusted analysis.

**Table 4:** Physiological markers and the reported or measured cardiometabolic problems.

Physiological marker/Exposure	Male				Female			
	Crude		Adjusted		Crude		Adjusted	
	Coefficient	95%CI	Coefficient	95%CI	Coefficient	95%CI	Coefficient	95%CI
IL-6 (pg/mL)								
Hypertension		p < 0.001		p = 0.450		p < 0.001		p = 0.148
No	1.16	1.12; 1.21	1.17	1.13; 1.22	1.33	1.28; 1.38	1.42	1.37; 1.47
Yes	1.32	1.24; 1.40	1.21	1.13; 1.29	1.74	1.58; 1.92	1.53	1.39; 1.66
Dyslipidemia		p < 0.001		p < 0.001		p < 0.001		p < 0.001
No	1.07	1.03; 1.12	1.08	1.03; 1.12	1.23	1.18; 1.29	1.35	1.29; 1.41
Yes	1.50	1.42; 1.58	1.40	1.32; 1.48	1.56	1.48; 1.63	1.53	1.46; 1.60
Diabetes		p = 0.486		p = 0.668		p = 0.255		p = 0.043
No	1.21	1.17; 1.25	1.19	1.14; 1.22	1.37	1.32; 1.42	1.42	1.38; 1.47
Yes	1.14	0.96; 1.35	1.14	0.94; 1.34	1.50	1.29; 1.75	1.65	1.42; 1.88
CRP (mg/L)								
Hypertension		p < 0.001		p = 0.307		p < 0.001		p = 0.178
No	0.66	0.62; 0.71	0.72	0.67; 0.78	1.54	1.44; 1.64	1.89	1.76; 2.02
Yes	0.85	0.76; 0.95	0.77	0.69; 0.86	2.38	2.00; 2.83	2.14	1.78; 2.50
Dyslipidemia		p < 0.001		p < 0.001		p < 0.001		p = 0.005
No	0.59	0.55; 0.63	0.64	0.59; 0.69	1.44	1.32; 1.56	1.76	1.61; 1.92
Yes	1.03	0.93; 1.13	0.92	0.83; 1.02	1.84	1.69; 2.01	2.09	1.91; 2.28
Diabetes		p = 0.844		p = 0.452		p = 0.255		p = 0.145
No	0.71	0.67; 0.76	0.73	0.69; 0.78	1.61	1.51; 1.71	1.90	1.78; 2.04
Yes	0.69	0.52; 0.92	0.83	0.58; 1.07	1.90	1.43; 2.53	2.33	1.71; 2.95
Adiponectin (µg/dL)								
Hypertension		p = 0.004		p = 0.244		p < 0.001		p = 0.208
No	8.15	7.92; 8.37	8.00	7.76; 8.25	10.87	10.64; 11.10	10.72	10.49; 10.95
Yes	7.52	7.16; 7.88	7.72	7.32; 8.12	9.65	9.02; 10.27	10.27	9.62; 10.93
Dyslipidemia		p < 0.001		p < 0.001		p < 0.001		p < 0.001
No	8.59	8.36; 8.82	8.46	8.21; 8.71	11.92	11.63; 12.21	11.67	11.37; 11.97
Yes	6.79	6.47; 7.11	6.88	6.53; 7.24	9.40	9.10; 9.70	9.55	9.23; 9.86
Diabetes		p = 0.438		p = 0.613		p = 0.654		p = 0.132
No	7.96	7.76; 8.15	7.92	7.71; 8.13	10.72	10.50; 10.95	10.70	10.48; 10.92
Yes	8.34	7.39; 9.29	8.19	7.15; 9.23	10.49	9.47; 11.50	9.90	8.88; 10.91

95%CI: 95% confidence interval; CRP: C-reactive protein; IL-6: interleukin 6.

Note: cardiometabolic problems included hypertension, dyslipidemia and diabetes. Adjusted for skin color, asset index at 22 years, fat mass index (dual-energy X-ray absorptiometry - DXA), difference of time between blood drawing and last meal, use of oral contraceptive, and behavioral variables. Regressions performed with IL-6 and CRP on logarithmic scale - results presented as exponential means and adiponectin as mean (95%CI). In bold: p-value < 0.0028 was considered statistically significant after Bonferroni correction.

## Discussion

Studies assessing physiological markers and multimorbidity are scarce, especially in young adults, but IL-6 and CRP seem to be good options for investigating this association ^12^. Adiponectin has been associated with lower adiposity levels and higher bone mineral density ^19^ ^,^ ^20^. IL-6 and CRP have already been linked to higher adiposity and mortality and lower bone mineral density ^14^ ^,^ ^19^ ^,^ ^20^.

As purposed, we found a physiological marker associated with multimorbidity in females, which had a higher prevalence of dyslipidemia than males. Overall, dyslipidemia consists of triglycerides, free fatty acids, HDL-cholesterol, and LDL-cholesterol abnormalities, being associated with a pro-inflammatory gradient, which may partially be originated in the adipose tissue ^31^. IL-6 and adiponectin are cytokines secreted by mature adipocytes. Adipose tissue is an active endocrine and paracrine organ that releases large amounts of cytokines and bioactive mediators, influencing body weight homeostasis via diverse metabolic processes ^32^. IL-6 is described as an inflammatory mediator and the main inducer of liver secretion of CRP, an acute-phase protein. IL-6 not only contributes to CRP elevation and low-grade inflammatory state but also has a close relation with coagulation, insulin resistance, dyslipidemia, and endothelial dysfunction ^33^. In its turn, adiponectin has several metabolic effects, including anti-inflammatory, insulin-sensitizing, and anti-atherogenic ^34^. Adiponectin strongly suppresses hepatic gluconeogenesis by inhibiting genes involved in glucose production, promoting insulin sensitization and, therefore, improving the whole body’s energy homeostasis ^35^. Furthermore, adiponectin induces interleukin 10 (IL-10) synthesis and acts against vascular inflammation ^36^, showing strong anti-inflammatory effects on cells such as macrophages and fibrogenic cells ^37^. Plasma adiponectin concentrations are influenced by several factors that regulate adiponectin gene expression and secretion, including IL-6 ^38^. Our findings showed a pro-inflammatory tendency in the presence of multimorbidity or disease accumulation, reinforcing the inverse direction of action between IL-6/CRP and adiponectin.

Furthermore, the associations between markers and multimorbidity or disease accumulation varied according to sex, being adiposity a probable link. In general, females have higher adiposity than males, although males have a higher incidence of metabolic diseases with the same BMI that females ^39^. It is necessary to consider the contribution of specific fat depots to cytokine secretion. Opposite influences of abdominal (visceral, deep subcutaneous, and superficial subcutaneous) and gluteal-femoral body fat in the modulation of circulating adiponectin were previously demonstrated ^40^. Also, higher adiponectin gene expression was described in subcutaneous than in visceral adipose tissue ^41^. Our findings showed higher adiponectin concentrations in females than in males, which agrees with the literature ^40^ ^,^ ^42^. Although we did not explore different adipose tissue depots, we adjusted for trunk fat mass. Therefore, we can suggest that our findings regarding adiponectin may be explained by higher gluteal-femoral depot in females, as they are at reproductive age.

Sex chromosomes and hormones are considered major determinants of sexual dimorphism in adiposity ^43^. Estrogens regulate key features of metabolism such as food intake, body weight, glucose homeostasis/insulin sensitivity, body fat distribution, lipolysis/lipogenesis, inflammation, locomotor activity, energy expenditure, reproduction, and cognition ^44^. Considering that, estrogens play a protective role, improving insulin resistance and dampening inflammation in obesity ^45^. The expansion of the gluteal-femoral depot driven by estrogens could prevent the development of metabolic dysfunction when facing an energy surplus. The immunometabolic links between estrogen, adipose tissue, and female reproductive metabolism have been recently discussed elsewhere ^43^. The inhibition of adiponectin production by circulating testosterone has also been described, which helps to understand the lower adiponectin concentration in males ^46^.

It is remarkable that females showed higher concentrations of all studied markers than males. Possibly, higher adiponectin concentrations physiologically exist to counteract inflammatory cytokines. On the other hand, in the presence of pathological settings as found in multimorbidity, the decrease in adiponectin concentrations indicates an imbalance in favor of inflammatory conditions related to dyslipidemia and cardiometabolic problems.

The prevalence of multimorbidity in young individuals highlights the impact of multiple chronic conditions especially cardiovascular problems since early adulthood. Note that the onset of morbidities probably occurred recently, implying a lack of more consistent associations because latency period of morbidities may not have been sufficient for pertinent rises in the physiological markers. In this context, most studies on similar associations were conducted with aging populations, which may justify different results as previously reviewed ^12^.

The study strengths are related to the way morbidities were identified, using objective measurements and validated instruments. Adjusted analyses were performed to decrease the influence of socioeconomic status, lifestyle, and adiposity on our findings.

### Limitations of this study

Limitations such as the use of medical diagnosis for some morbidities should also be highlighted, as it is influenced by access to health services and tends to be lower in individuals from lower socioeconomic strata. Another limitation is the lack of standardization in the definition of multimorbidity, which represents a challenge for comparisons among studies. Finally, this investigation corresponds to a cross-sectional analysis that represents data from a specific point in time. We know systemic inflammation is considered a risk factor for several diseases, including cardiovascular disease, but it can also be a product of pathology. There is also a possibility that adiposity is part of the multimorbidity causal chain, thus we adjusted our analyses for this variable, which might have weakened the associations. Therefore, this study design does not enable establishing inferences about causality. This article is intended to be a starting point for future follow-up analyses, so further longitudinal studies are needed to better understand these associations.

## Conclusion

Our findings suggest an inverse association between multimorbidity and adiponectin in females, as well as a direct cumulative association between the number of diseases and IL-6 and CRP in males. The analysis of a young population suggests the effect of morbidities accumulation on markers may be explained by dyslipidemia. Further cohort studies with longer latency periods are necessary to determine the direction of associations and influence of the accumulation of different morbidities on physiological markers.
