# Supplementary material for: Association of inflammatory markers and multimorbidity in young adults: cross-sectional findings from the Pelotas (Brazil) birth cohort, 1993
Source: Cad Saude Publica. 2025 Feb 7;41(1):e00191623. doi: 10.1590/0102-311XEN191623 (PMC11805519; doi:10.1590/0102-311XEN191623)
Supplement: Supplementary file 1 [file 1678-4464-csp-41-01-EN191623-s.pdf]

Supplementary Material

Panel A

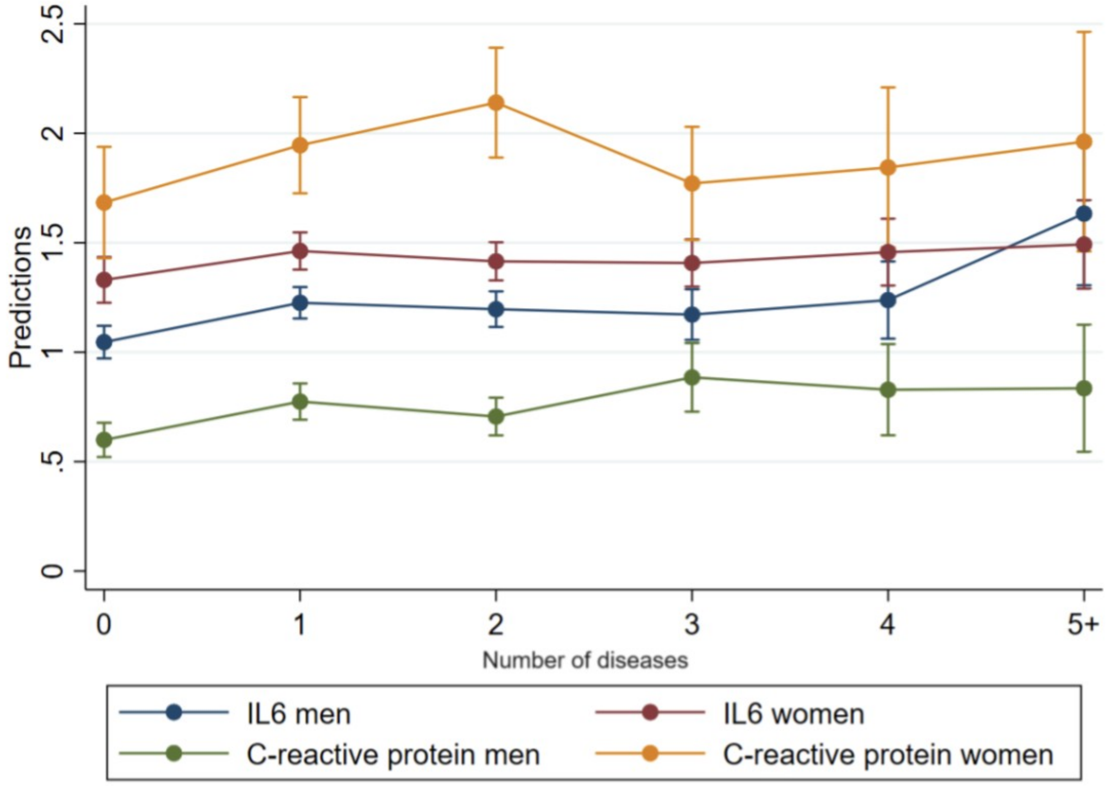

Panel B

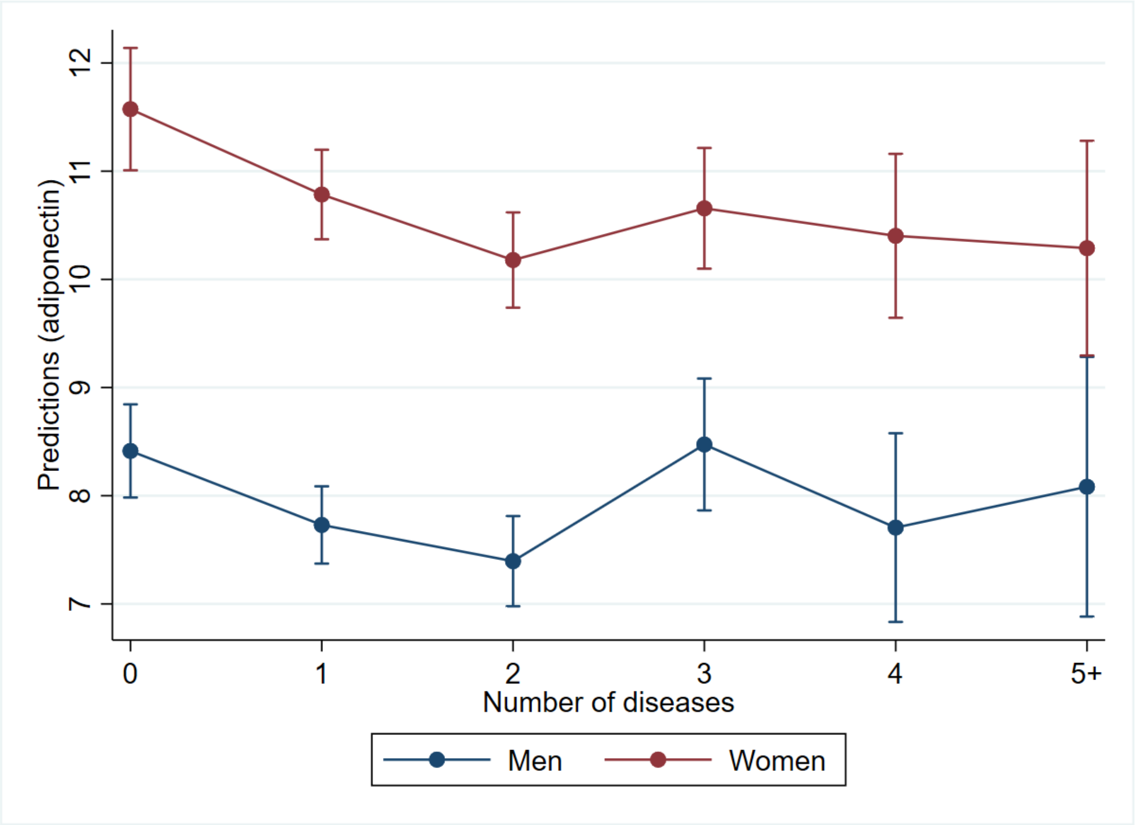

Additional supporting file. Adjusted regression analyses between markers and number of diseases (0 to 5+) stratified by sex.

Panel A: *P*-values by the Wald's test for linear tendency for IL-6 were equal to 0.005 in men and 0.279 in women. For CRP, P-values were 0.006 in men and 0.272 in women. After Bonferroni correction a P-value <0.0125 was considered significant in both cases.

Panel B: The *P*-values by the Wald's test for linear tendency from the regression analysis between adiponectin and number of diseases were 0.009 and 0.005 in men and women, respectively. After Bonferroni correction a P-value <0.0025 was considered significant.

Figure S1. Analysis of the distribution of C-reactive protein with graphs before and after logarithmic transformation, respectively.

```
. sum lpcr_pcr,d

                     proteina C-reativa
-------------------------------------------------------------
      Percentiles      Smallest
 1%           .1              0
 5%           .1             .1
10%           .2             .1       Obs               3,481
25%           .4             .1       Sum of Wgt.       3,481

50%            1                      Mean           2.928699
                        Largest       Std. Dev.      7.062931
75%          2.7           86.5
90%          6.6           87.9       Variance         49.885
95%         11.3          118.5       Skewness       9.313652
99%         29.3          163.6       Kurtosis       139.1258
```

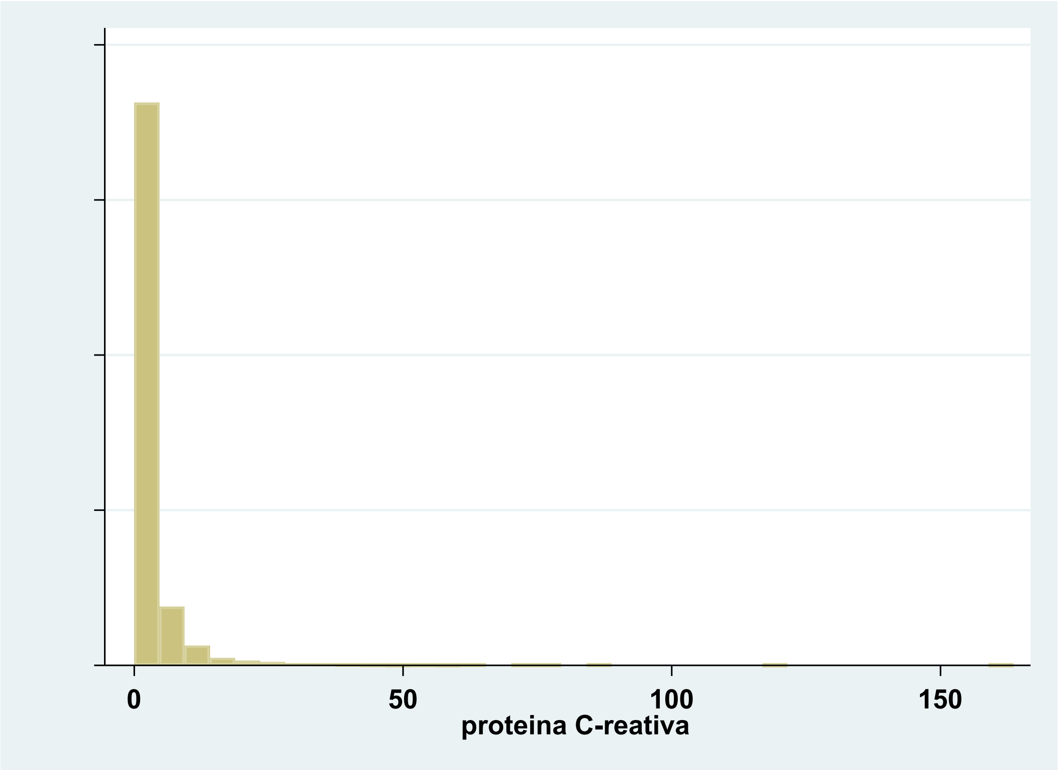


```
. sum lnpcr,d

                            lnpcr
-------------------------------------------------------------
      Percentiles      Smallest
 1%    -2.302585      -2.302585
 5%    -2.302585      -2.302585
10%    -1.609438      -2.302585       Obs               3,480
25%    -.9162907      -2.302585       Sum of Wgt.       3,480

50%            0                      Mean           .0877619
                        Largest       Std. Dev.      1.339958
75%     .9932518       4.460145
90%      1.88707         4.4762       Variance       1.795487
95%     2.424803       4.774913       Skewness       .3271137
99%     3.377588       5.097425       Kurtosis       2.780794
```

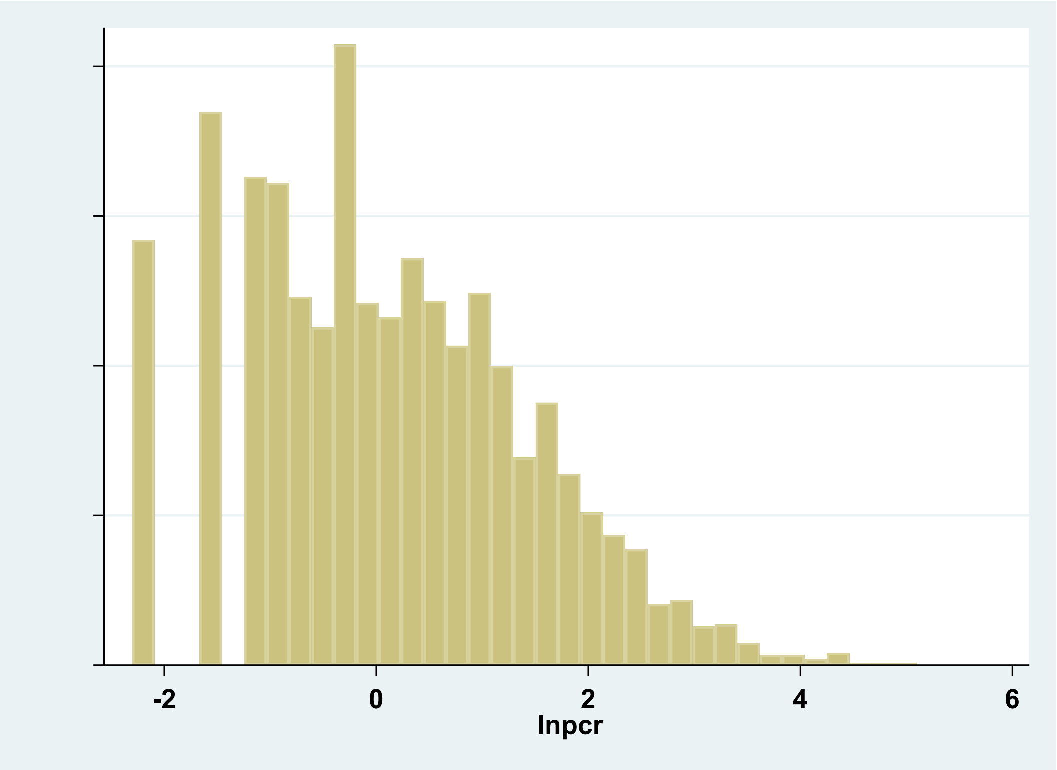

Figure S2. Analysis of the distribution of interleukin-6 with graphs before and after logarithmic transformation, respectively.

```
. sum lAvgconc22,d

                        concentra□□o media IL-6
-------------------------------------------------------------
      Percentiles      Smallest
 1%         .394           .271
 5%         .525           .297
10%           .6            .31       Obs               3,138
25%          .81           .315       Sum of Wgt.       3,138

50%       1.1625                      Mean           1.701009
                        Largest       Std. Dev.      1.840678
75%        1.842         19.947
90%        3.222         20.728       Variance       3.388096
95%        4.519         20.878       Skewness       4.775192
99%       10.289         22.283       Kurtosis        35.9656
```

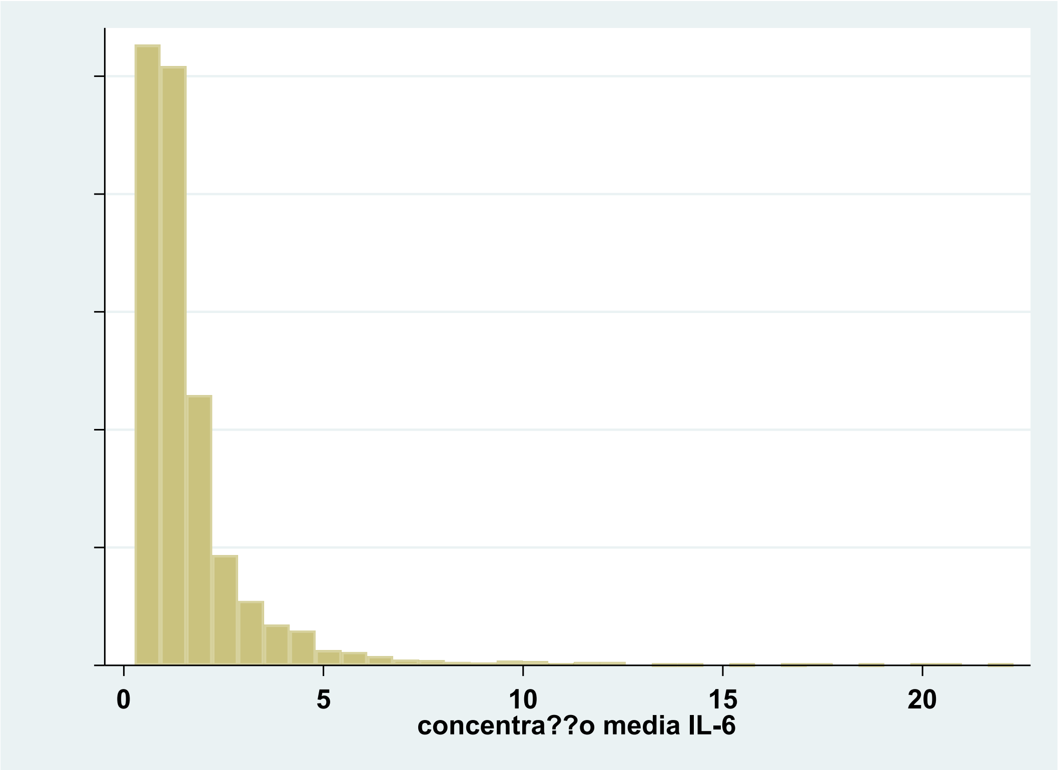


```
. sum lnil6,d

                            lnil6
-------------------------------------------------------------
      Percentiles      Smallest
 1%    -.9314044      -1.305636
 5%     -.644357      -1.214023
10%    -.5108256      -1.171183       Obs               3,138
25%     -.210721      -1.155183       Sum of Wgt.       3,138

50%     .1505728                      Mean           .2531317
                        Largest       Std. Dev.      .6706931
75%     .6108519       2.993079
90%     1.170002       3.031486       Variance       .4498293
95%     1.508291       3.038696       Skewness       .8893101
99%     2.331075       3.103824       Kurtosis       4.085729
```

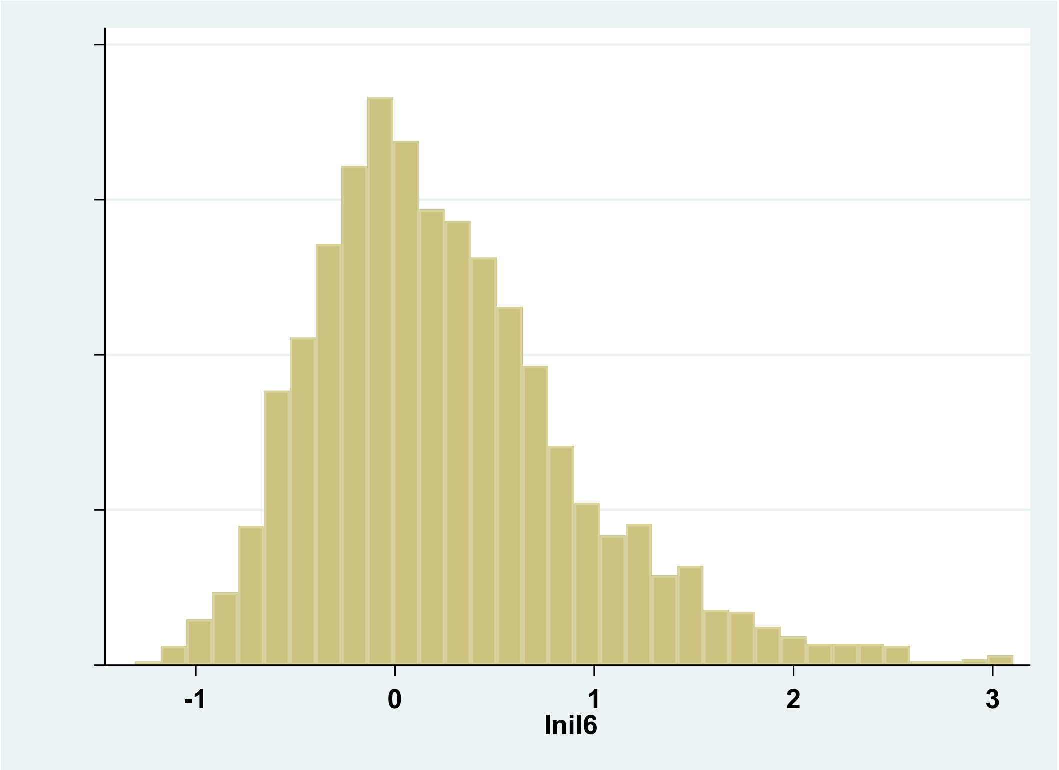

-1
0
1
2
3
lnil6
